# Draft Genome Sequence of the Cyanotroph Pseudomonas monteilii BCN3

**DOI:** 10.1128/MRA.01322-18

**Published:** 2019-01-03

**Authors:** Lauren B. Jones, Daniel A. Kunz

**Affiliations:** a Department of Biological Sciences, Division of Biochemistry and Molecular Biology, University of North Texas, Denton, Texas, USA; University of Arizona

## Abstract

We report here the first draft genome of Pseudomonas monteilii BCN3, a cyanotroph isolated from sewage sludge. The genome consists of approximately 6,029,517 bp with a G+C content of 61.89% and 5,369 annotated protein-coding genes.

## ANNOUNCEMENT

Pseudomonas monteilii (formerly Pseudomonas putida) BCN3, isolated as previously described (1), exhibits the ability to grow in minimal medium (2) with cyanide as the sole nitrogen source (cyanotrophy). Of 10 separate bacteria previously isolated, P. monteilii BCN3 exhibited the fastest growth rate (generation time [t_gen_], 2 h) and was therefore chosen for genome sequencing for the purpose of uncovering unique genes linked to cyanide growth. Here, we report the draft genome sequence of the first representative P. monteilii strain able to grow cyanotrophically.

Genomic DNA was isolated employing an UltraClean microbial DNA isolation kit (Mo Bio, Qiagen) according to the manufacturer’s instructions. Cells of P. monteilii BCN3 were inoculated from a single colony and grown overnight at 30°C in complete medium (Lennox broth [LB]) (3). Genomic DNA was prepared using the Illumina Nextera DNA Flex library preparation kit, and a 76-bp insert library was sequenced at the University of North Texas BioDiscovery Institute Genomics Center using Illumina NextSeq system technology. This produced 6,451,536 paired-end reads with an average read length of 76 bp (81.4× coverage) that were quality checked using FastQC version 1.0.0 with default parameters (BaseSpace Labs, Illumina). The genome was *de novo* assembled using the SPAdes genome assembler version 3.9.0 (4) and improved through closing gaps with Rescaf version 1.0.1 (BaseSpace Labs, Illumina), in each case employing default parameters. A BLASTn search (5) using full-length P. monteilii BCN3 16S rRNA as a query (identified from a preliminary annotation by Rapid Annotations using Subsystems Technology [RAST]) (6) returned closely related P. monteilii and P. putida strains. The strain most similar to P. monteilii BCN3 for which the complete genome sequence is available, *Pseudomonas* sp. strain XWY1 (NCBI accession no. CP026332), was used as a reference to reorder the scaffolds of P. monteilii BCN3 DNA employing progressiveMauve version 2.3.1 (7) set to default parameters. The P. monteilii BCN3 genome was assembled into 96 scaffolds of 105 contigs (*N*_50_ value, 95,637 bp) with a total length of 6,029,517 bp and a G+C content of 61.9%. The NCBI Prokaryotic Genome Annotation Pipeline version 4.6 (8) was then used to predict and annotate P. monteilii BCN3 open reading frames, consisting of 5,369 protein and 81 RNA coding genes.

Sequencing the genome of P. monteilii BCN3 enabled genome comparisons for identifying genes linked to cyanotrophy. One such set of genes found conserved in other cyanotrophs (2, 9) is a seven-gene cluster known as Nit1C, shown here also to be present in P. monteilii BCN3 as identified from the annotation of the Nit1C genes (locus tags D0894_12380 to D0894_12410). A search of available bacterial genomes for Nit1C reveals its presence in 270 different bacteria, suggesting that additional cyanotrophs remain to be uncovered (2). Further identification of gene functions related to cyanotrophy are currently in progress.

### Data availability.

The draft P. monteilii BCN3 genome sequence has been deposited in DDBJ/ENA/GenBank under the accession no. QWLL00000000. The version described here is the first version of the draft genome. The Sequence Read Archive (SRA) accession no. is SRX4980437.
